# Recognizing the Importance and Impact of Academic Medicine

**DOI:** 10.31486/toj.23.5032

**Published:** 2023

**Authors:** Ronald G. Amedee

**Affiliations:** Director of Clinical School and Professor, The University of Queensland Medical School, Ochsner Clinical School, New Orleans, LA; Editor-in-Chief, *Ochsner Journal*

*There are two great days in a person's life—the day we are **born** and the day we **discover why***.

*–William Barclay*

Five original research articles, 2 reviews and contemporary updates, and 7 case reports serve as the principal elements for the Spring 2023 issue of the *Ochsner Journal*. In addition, we are pleased to introduce a new quarterly column—Health, Medicine, and Society—that will be facilitated by Dr Kevin Conrad and authored by Ochsner clinicians and practitioners from other institutions. In this debut column, Dr Conrad addresses the impact of climate change on heath care and suggests some strategies for mitigating the current challenges and preparing for the future. The spring issue also contains an editorial, “Physician Well-Being and the Promise of Positive Psychology,” and a letter to the editor, “Effect of Strabismus and Amblyopia on Postural Stability.”

Orthopedics/sports medicine is well represented in this issue's original research articles. Researchers at Anne Arundel Medical Center in Annapolis, Maryland, evaluated the impact of adductor canal block (ACB) on postoperative outcomes for patients undergoing total knee arthroplasty and found that ACB has little to no significant impact on early clinical outcomes.

Ochsner teams led by Dr Misty Suri examined the effect of body mass index (BMI) on hip arthroscopy outcomes and also evaluated the postoperative pain associated with bone-patellar tendon-bone anterior cruciate ligament reconstruction. In the first study, the team found no statistically significant effects of BMI on patient outcome scores following hip arthroscopy. For the second study, Suri et al define a novel outcome variable—functional anterior knee pain—that they maintain is a better measure of postoperative functional limitations than measures such as the knee-walk test. Three years postoperatively, 94% of the patients in the study were not limited by functional anterior knee pain and had returned to their preoperative levels of activity and sport.

Another Ochsner team led by Dr Leslie Sisco-Wise provides a 4-year retrospective cohort study of patients who underwent secondary open revision carpal tunnel release surgery with umbilical cord allograft. Revision surgery is often unsuccessful, but Dr Sisco-Wise and her group found that using human umbilical cord allograft improved long-term symptoms of compressive neuropathy.

Rounding out our original research articles is “Stop the Divide: Facilitators and Barriers to Uptake of Digital Health Interventions Among Socially Disadvantaged Populations” by Price-Haywood, Arnold, Harden-Barrios, and Davis that details the results of a Pfizer-sponsored study focused on telemedicine.

Reviews in this issue include a “Brief History of Opioids in Perioperative and Periprocedural Medicine to Inform the Future” that explores the history of opioid use in perioperative care from the mid 19th century to the present day and presents current research on the physiology of addiction. The other review, from an orthopedics team at LSU-Shreveport, examines the influence of COVID-19 on physician burnout and proposes methods to reduce work exhaustion for those in the health care field.

The dramatic cover images for the Spring 2023 issue are taken from a case report submitted by an Ochsner-Tulane plastic surgery team: “Two-Stage Pediatric Ear Reconstruction Using Preserved Native Cartilage After a Dog Bite.” The issue's orthopedic theme is continued with “Trigger Wrist Caused by a Rheumatoid Nodule on the Flexor Longus Tendon.” Other cases in this issue report 3 rare occurrences of different types of cancer and a fascinating case of a patient with bilateral agenesis of the internal carotid artery. The absence of the bilateral carotid canals and intracranial internal carotid arteries was discovered on imaging when the patient presented with sudden onset right eye pain radiating to her neck.

For many of us in academic medicine, we discover *why we were born* as we mentor, instruct, supervise, evaluate, and interact with learners at all levels in the continuum of medical education. The training of our future clinicians, researchers, and academicians in medicine is one of the most gratifying, noble, and impactful activities we can involve ourselves in as we progress through the 3+ decades of our respective careers. The time we spend educating them matters—to our learners, to our patients, and to each of us. Health care systems nationwide are currently facing financial crises for numerous reasons that are beyond the scope of this introduction. However, as clinical demands increase and additional RVUs are sought, we must not lose sight of our academic culture and must seek every opportunity to emphasize and reinvigorate our collective efforts to prioritize the needs of our learners. The excellence of health care in this country and beyond depends on us and ultimately on today's trainees.

